# Parabens Permeation through Biological Membranes: A Comparative Study Using Franz Cell Diffusion System and Biomimetic Liquid Chromatography

**DOI:** 10.3390/molecules27134263

**Published:** 2022-07-01

**Authors:** Ilaria Neri, Sonia Laneri, Ritamaria Di Lorenzo, Irene Dini, Giacomo Russo, Lucia Grumetto

**Affiliations:** 1 Department of Pharmacy, School of Medicine and Surgery, University of Naples Federico II, Via D. Montesano, 49, I-80131 Naples, Italy; ilaria.neri@unina.it (I.N.); slaneri@unina.it (S.L.); ritamaria.dilorenzo@unina.it (R.D.L.); irdini@unina.it (I.D.); 2 School of Applied Sciences, Sighthill Campus, Edinburgh Napier University, 9 Sighthill Ct, Edinburgh EH11 4BN, UK

**Keywords:** parabens, investigative toxicology, skin, Franz cell, lipophilicity, chromatography approach, immobilized artificial membrane

## Abstract

Parabens (PBs) are used as preservatives to extend the shelf life of various foodstuffs, and pharmaceutical and cosmetic preparations. In this work, the membrane barrier passage potential of a subset of seven parabens, i.e., methyl-, ethyl-, propyl- isopropyl, butyl, isobutyl, and benzyl paraben, along with their parent compound, p-hydroxy benzoic acid, were studied. Thus, the Franz cell diffusion (FDC) method, biomimetic liquid chromatography (BLC), and in silico prediction were performed to evaluate the soundness of both describing their permeation through the skin. While BLC allowed the achievement of a full scale of affinity for membrane phospholipids of the PBs under research, the permeation of parabens through Franz diffusion cells having a carbon chain > ethyl could not be measured in a fully aqueous medium, i.e., permeation enhancer-free conditions. Our results support that BLC and in silico prediction alone can occasionally be misleading in the permeability potential assessment of these preservatives, emphasizing the need for a multi-technique and integrated experimental approach.

## 1. Introduction

Parabens (PBs) are a group of C-4 esterified molecules of hydroxybenzoic acid (pHBA) with a broad antimicrobial and antifungal spectrum, commonly used since the 1920s [1] as preservatives in foodstuffs, pharmaceuticals, and personal care products (PCPs) to increase their shelf life [2,3]. PBs having a higher value of n-octanol–water partition coefficient (log P ranging from 1.96 to 3.57) show low water solubility, making the shorter-chained PBs better suited for their application. These compounds can leak into the environment because they are massively employed in the industry and released mainly through wastewater treatment discharges [2]. PBs can be easily absorbed by the human body, both orally and after dermal or respiratory exposure [4]. These are stable in the bloodstream for 24h and then are metabolized, mainly by carboxylesterases, releasing nonspecific pHBA and p-hydroxy hippuric acid and, less frequently, are excreted to various molecules as free, oxidized, or conjugated in the microsomes [5]. Some non-metabolized parabens bioaccumulate in the various compartments of the human body [6,7]. In addition, these toxicants can cross the placenta barrier, leading to potential fetal exposure [8].

PBs act by altering mitochondria and membrane transport, with their long-chain ester group showing higher antimicrobial properties. Moreover, the longer-chained PBs, such as Butyl 4-hydroxybenzoate (BuP) and Propyl 4-hydroxybenzoate (PrP), show higher endocrine disruptive properties than short-chain analogs, such as Methyl 4-hydroxybenzoate (MP) [9,10]. In recent decades, numerous studies aimed at assessing the effects of PBs on the endocrine system, and currently, an ample body of scientific literature, indicate that these can exert estrogenic activity and can therefore be considered as endocrine disrupting compounds (EDCs) featuring a non-steroidal chemical structure [11,12,13,14,15]. Indeed, several studies confirm the hypothesis that PBs can act as EDCs that can modulate the functions of the endocrine system [16,17,18]. Some of them, MeP, EtP, PrP, and BuP, competitively bind the ERs [13]) and impact ER-dependent gene expression [19,20], interfering in the normal functioning of natural endocrine hormones.

Moreover, PBs can affect reproduction by altering fertility and reproductive functions in male rodents after repetitive oral exposure by causing epigenetic hypermethylation of sperm DNA, which may impact transcription regulation and be transmitted to the offspring [21]. Although their acute toxicity is low [22], a gap in knowledge subsists about their health effects after chronic exposure, which currently prevents an accurate risk assessment. This work aimed to explore the passage of seven parabens and their parent compound, pHBA, through the skin, the most prevalent route of human exposure [23], by measuring (a) their permeability using a traditional Franz diffusion cell system and (b) their affinity for membrane phospholipids by immobilized artificial membrane (IAM) liquid chromatography (LC).

**Figure 1:**
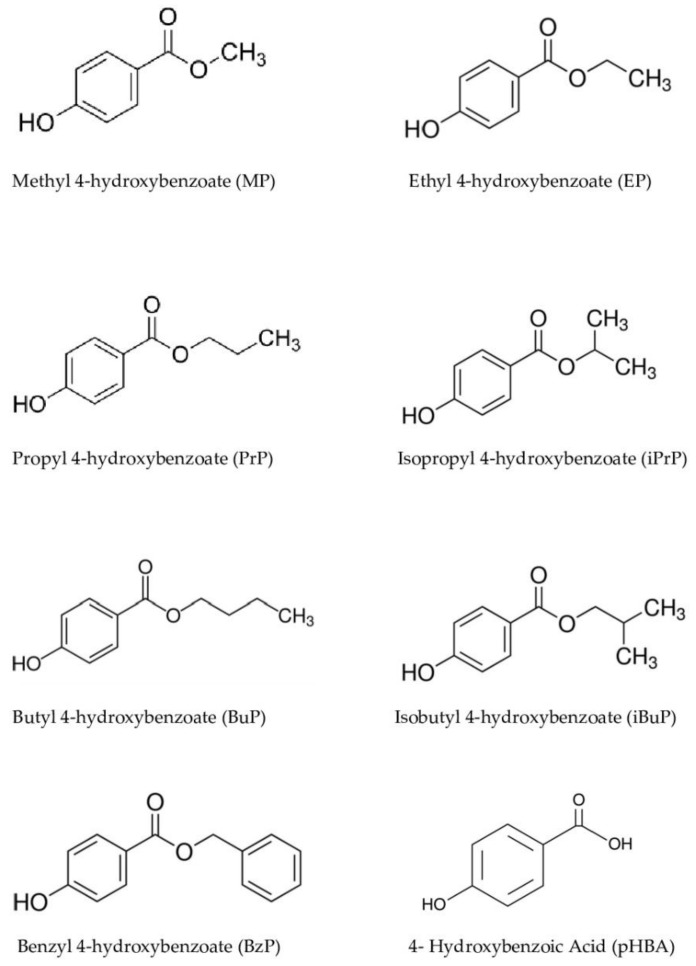
Chemical structure of parabens under investigation.

Figure 1 shows the chemical structures of analytes we investigated: Methyl 4-hydroxybenzoate (MP), Ethyl 4-hydroxybenzoate (EP), Propyl 4-hydroxybenzoate (PrP), Butyl 4-hydroxybenzoate (BuP), Isopropyl 4-hydroxybenzoate (iPrP), Benzyl 4-hydroxybenzoate (BzP), Isobutyl 4-hydroxybenzoate (iBuP), and pHBA. Some PBs, i.e., MP, EP, BuP, PrP, and pHBA, are authorized in PCPs, up to 0.4% for a single ester and 0.8% in mixtures. Isopropyl-, isobutyl-, phenyl-, benzyl-, and pentylparabens have been banned from use in PCPs in Europe since 2014 [24].

Immobilized artificial membrane stationary phases consist of phosphatidylcholine (PC) analogs covalently bound to silica, aiming to mimic biological cell membranes closely. The degree of affinity between analytes and the IAM stationary phases is regarded as phospholipophilicity and indicated by the logarithm of coefficient of analytical retention achieved at or extrapolated to 100% aqueous conditions (log kw IAM). Indeed, previous studies by our research team demonstrated a good correlation between data obtained with traditional in vitro methods (intestinal tissues and passage through cell bilayers) and those achieved with this analytical technique [25,26], which was proven to be effective in studying the membrane permeability potential of target compounds both in vivo and in situ [27,28]. Analogously, we decided to explore the possible relationships between BLC and transdermic passage in order to verify if the lipophilicity can predict the PBs’ permeation.

## 2. Results and Discussion

This work aimed at studying the membrane affinity and the dermal absorption of seven parabens—some of which, i.e., MP, EP, BuP, PrP, are allowed in PCPs—and their parent compound, pHBA. Table 1 summarizes chromatographic parameters applied to determine the analytes in the acceptor chambers of the FDC, the calibration curve parameters, and relevant physicochemical properties. Chromatographic conditions used in HPLC-UV methods for the quantification of all eight chemicals in receptor fluid solution were found sensitive (up to 2.5 μg mL^−1^) to their detection, and a sound linear relationship (r^2^ > 0.9256) was found between the instrument response and the applied concentration values spanning in the range 0.052–0.886 µg mL^−1^ (LOD values in Table S1).

Permeation results, as assessed by Franz cell diffusion study, are shown in Table 2 along with the percentage permeation consistent with skin permeation values.

MP and pHBA exhibited a maximum passage through the porcine skin within 30 min from the beginning of the experiment, while EP passage was realized within 120 min. Furthermore, as shown in Table 2, the EP Kp value was lower than MP’s. In fact, MP was found to have a maximum flux value of 76.23 ± 26.60 (µg/cm^2^/h), which is significantly higher than that of EP (maximum flux of 2.35 ± 0.71 µg/cm^2^/h). Thus, PBs with a shorter alkyl chain present higher permeation, MP > EP, and show that the skin permeation degree of PBs can be considered moderate, according to the definition of Marzulli et al. [29]. Conversely, starting from PrP, analogs with longer chains were not detected in receptor fluids at any time. The pHBA showed an intermediate passage profile between MP and EP, probably due to its free ionizable carboxyl acid group with a pKa of 4.01 (SwissADME), which is partially ionized at the experimental pH (5.0).

Over the years, in vitro Franz diffusion experiments have evolved into one of the most important methods for researching transdermal absorption [30]. However, these procedures have some shortcomings, including their being time-consuming and poorly inter-lab reproducible. For this reason, an increased demand for higher throughput, more inter-lab reproducible methods emerged. Chromatography performed on stationary phases emulating the asset of biological membranes cannot directly describe the permeation of the analytes but can accidentally assist in the pharmacokinetic (PK) profiling of an array of target compounds and potentially offer usefulness in surrogating permeation data [31].

Table 3 shows experimental log kw IAM performed on both columns, IAM.PC.MG and IAM.DD2.

**Figure 2:**
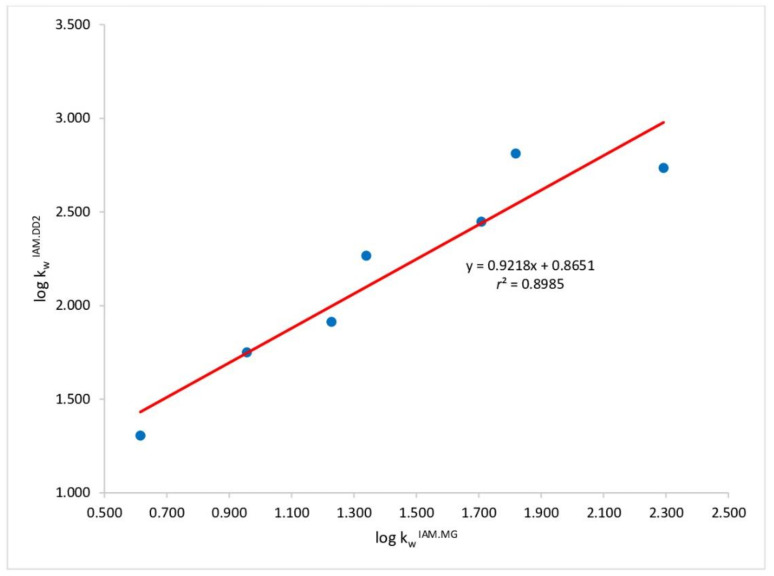
Relationship with phospholipophilicity data achieved on IAM.PC.MG and IAM.PC.DD2 stationary phases.

A good relationship (r^2^ = 0.898) was found between log kwIAM values determined on the IAM.PC.MG and IAM.PC.DD2 stationary phases, as can be seen in Figure 2, suggesting that the role of the end-capping in the analytical retention is only marginal.

In the Supplementary Materials (Table S2 and Figure S1), we also show the in silico calculated clogkw IAM.MG vs. clogkw IAM.DD2 values having a similar trend but are slightly higher than the experimental values. These are achieved via a user-friend Web service (available at https://nova.disfarm.unimi.it/vegaol/logkwiam.htm (accessed on 27 June 2022) able to predict the phospholipophilicity of any molecule included in the PubChem collection [28]. Table 4 shows Kp values achieved by SwissADME^®^ software (Swiss Institute of Bioinformatics^©^ 2022, Lausanne, Switzerland) compared to those for the PBs able to cross the skin in our experimental conditions.

**Figure 3:**
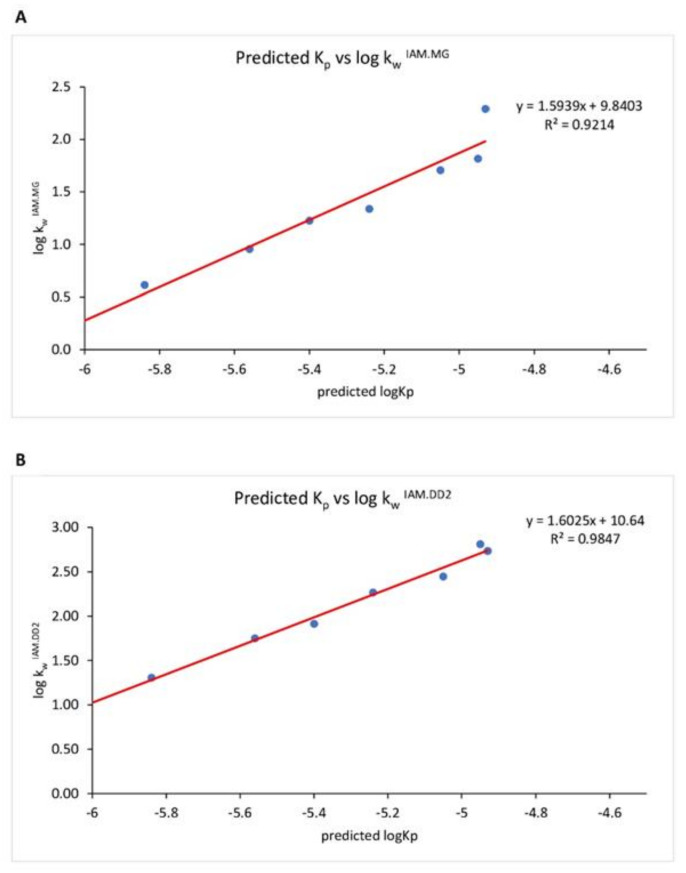
Relationship between Log Kp values achieved from SwissADME^®^ software and log kw ^IAM.MG^ (**A**) and logkw ^IAM.DD2^ (**B**) values achieved by IAM chromatography. A good relationship is observed, demonstrating that IAM experimental data of affinity for phospholipid agree with skin permeation data.

In Figure 3, we report the relationship between log kwIAM.MG (a) and log kwIAM.DD2 (b) values achieved for all chemicals under investigation but pHBA, already reported in Table 3, versus Log Kp values achieved from SwissADME^®^ software. Indeed, pHBA is the only acidic analyte of the dataset. According to the pH piston hypothesis formulated by Avdeef [32], after some experimental observations about the partitioning of drugs into liposomes, negatively charged molecules interact with the positively charged ammonium groups of PC moieties located at the outer side of the membranes, engage more externally, and remain on the surface. In contrast, cations interact with the negatively charged phosphate moieties that are located inside. Consistently, pHBA, as a carboxylate at experimental pH 5.0, is less retained by both IAM phases than a neutral isolipophilic molecule and therefore exhibited a log kw IAM value < 1. When we compare IAM data with in silico permeation values achieved by the software SwissADME^®^, a good relationship is observed if pHBA is left out (Figure 3). Indeed, phospholipophilicity, which effectively describes the affinity of chemicals to the membrane [33], can be used in combination with other methods to shed light on the molecular features of these preservatives driving their toxic effects and specifically their endocrine-disrupting properties.

Similar to SwissADME^®^ software, many in silico methods share the objective of predicting the penetration of molecules through the skin, and these normally exploit quantitative structure–property relationships (QSPRs), models based on diffusion mechanisms, or a combination of both [34]. Mathematical tools consist of equations to predict skin permeability based on physicochemical properties, such as molecular weight and the octanol-water partition coefficient (log Kow) [35,36,37,38,39]. The Kp data achieved by SwissADME^®^ contradict the experimental permeation data. Indeed, according to the in silico data, the Kp values would be rather similar for the whole dataset, and there is only a slight difference in permeation rate starting from MP up to the higher chain analog. Instead, in our FDC experiment, only three compounds could cross the pig skin membrane.

We should highlight that membrane crossing is a complex phenomenon where multiple passage pathways that cannot be necessarily accounted for by either in silico or IAM methods can occur. The routes of permeation of the molecules across the skin includes the transcellular route where the molecule encounters the low lipid regions in the cytoplasm of the corneocytes; the intercellular route of dense lipids and fatty acids featuring both hydrophilic and lipophilic regions, making this route very resistant to permeation; and the shunt pathway or appendageal route with diffusion into the sweat gland, hair follicles, and sebaceous gland [40]. Indeed, the diffusion of the target molecule through the lipoidal layer is regarded as transcellular passage, and that is the mechanism that most compounds of pharmaceutical/environmental concern seem to exploit. However, smaller hydrophilic molecules can overcome the skin barrier by passing through the intercellular space between the cells, therefore using the intercellular route [41]. The relative contribution in the overall passage depends mainly (but not exclusively) upon the molecular volume. It is reasonable to assume that the contribution of the intercellular passage for MP is greater than that of EP for the relatively lower molecular weight and, therefore, molecular volume.

Our FDC data substantiate that the permeation potential of the target preservatives could also be affected by solubility, which decreases at increasing chain length, and so, the absolute amount exposed to the membrane for PBs with chain length > propyl is fairly inferior to that of other PBs [42]. The excipients contained in various formulations may potentially affect the skin crossing [43,44,45] by

- Decreasing the polarity of the medium and increasing the relative solubility of our target preservatives, and
- Disrupting the packing of lipids into biological membranes and increasing their leakiness.

For these reasons, most of these excipients can act as enhancers to increase skin barrier permeability. Indeed, in the Caon et al. study [42], PBs are dissolved in ethanol, a well-known promoter of skin passage [46], at various percentages (0.1% paraben dissolved in an ethanol/PBS mixture—20:80 and 50:50), while other studies introduced PBs in donor chambers as cream (O/W emulsion) formulations [47] or as commercial body lotions [48]. For instance, Hatami et al. evaluated the passage of MP and BuP from an emulsion using the FDC system, observing that MP has lower permeation and lower extent than BuP, adding PBs in the oily phase of the formulation [49]. Conversely, our experiments were run in a fully aqueous phase at pH 5.0, enhancer-free conditions, with saturated solutions of each PB, and thus, our results are partially consistent with those other previously mentioned studies, since only MP and EP and not PrP and BuP were found in receptor fluids. We underline that the design of the FDC experiments was underpinned to study the skin passage of the PBs per se, not as ingredients of a formulation because even if the employed formulations contain enhancer excipients, a 100% aqueous vehicle is the most appropriate standard state to assess the permeation [39].

Lipophilicity is a driving force of the transmigration of xenobiotics through the membrane, but the presence of other active/excipients can reduce surface tension, minimizing its impact on the overall permeation.

For this reason, the surrogate models such as in silico prediction and BLC should only be used as preliminary screening tools for chemicals to be followed by diffusion studies exploiting natural skin model, as these can fail in the estimation of molecule’s permeation.

## 3. Materials and Methods

### 3.1. Chemicals

Methyl 4-hydroxybenzoate (MP), Ethyl 4-hydroxybenzoate (EP), Propyl 4-hydroxybenzoate (PrP), and Butyl 4-hydroxybenzoate (BuP) were purchased from Merck & Co. (Poole, UK). Isopropyl 4-hydroxybenzoate (iPrP) was purchased from Fluorochem (Hadfield, UK), Benzyl 4-hydroxybenzoate (BzP) was purchased from Sigma Aldrich (Milan, Italy), and Isobutyl 4-hydroxybenzoate (iBuP) and 4-Hydroxybenzoic acid (pHBA) were purchased from J&K Scientific (San Jose, CA, USA). The purity of all PBs was equal to or higher than 98%. Milli-Q water was produced in-house, and its conductivity was 0.055 μS cm^−1^ at 25 °C (resistivity equals 18.2 MΩ·cm). Phosphate buffered saline (PBS) was freshly made in-house by adding disodium hydrogen phosphate and sodium chloride (J.T. Baker, Phillipsburg, NJ, USA), and potassium chloride (Sigma Aldrich), by diluting with Milli-Q water to 1 L. The pH (7.00) was determined by a pH meter. Acetonitrile (ACN) and methanol (MeOH) were HPLC grade and purchased from Sigma Aldrich (Milan, Italy).

### 3.2. Tissue Preparation

For the ex vivo skin penetration studies, the Organization for Economic Co-operation and Development (OECD) guideline recommends pig ear skin as a suitable skin surrogate to mimic human percutaneous penetration [24]. The skin samples were excised from a male pig ear, post-sacrifice, obtained from a local abattoir (Avellino, Italy) within three hours from animal death. The outer part of the ear was used.

The integrity of the skin area used for the permeation experiment was examined by measuring non-invasive trans-epidermal water loss (TEWL) using an In-vitro Tewameter^®^ VT 310^®^ (Courage + Khazaka electronic GmbH, Köln, Germany), and only the skin samples with TEWL values < 10 g/m^2^/h were used in this study. Subcutaneous fat was removed, and skin samples were kept at room temperature and hydrated in saline solution (0.9% NaCl) for 5 min. Then, skin specimens were put on a filter paper (Fisherbrand™ Grade 601, Fisher Scientific, Leicestershire, UK) and cut into pieces before assembly in the Franz Diffusion Cell (FDC).

### 3.3. Skin Permeation

Skin absorption tests were performed using Franz-type vertical static diffusion cells (FDC Ø 9 mm, 5 mL receptor compartment, SES GmbH-Analyse System, Bechenheim, Germany) with an effective diffusion area of 0.6 cm^2^ and receptor volume of 5.0 mL [50]. The total thickness of the skin was washed three times with 1.0 mL of water and mounted between the two halves of the cell with the stratum corneum facing the donor compartment. The receptor compartment was filled with 5.0 mL of PBS pH 7.4 and checked to ensure there were no air bubbles between the receptor fluid (PBS) and the skin. PBS is a buffer solution that is particularly valuable because it mimics the ion concentration, osmolarity, and pH of human body fluids [51]. The donor compartment was filled with 1 mL of a saturated solution (concentration values are listed in Table 1) of each analyte dissolved in PBS pH 5.0. Two cells were added as blanks filled with only PBS pH 5.0 as donor solutions. The experiments were repeated in triplicate for a total of 24 exposed cells and two blanks. The FDC was maintained at a constant temperature of 37 ± 0.5 °C through thermostatic bath circulation, and the receptor medium was constantly kept under magnetic stirring throughout the experiments using a Teflon cylindrical magnetic stirring bar (dimension 10 × 3 mm). At fixed time intervals (½, 1, 2, 3, 4, 5, 6, and 24 h), aliquots were collected from the receptor chambers and immediately replaced with the same volume of fresh PBS medium. The collected samples were analyzed by high-performance liquid chromatography (HPLC) in triplicate to quantify the amounts of each PB.

### 3.4. Chromatographic Analysis

PBs’ quantification was obtained by the regression equation obtained from standard curves prepared on the same day and performed in concentration levels according to the solubility of each PB to achieve a concentration close to saturated solution. Analytical methods were set up ad hoc to analyze chemicals of interest in the receptor’s fluids. Analyses were performed at room temperature (22 ± 2 °C). HPLC (LC-20 AD VP; Shimadzu Corp., Kyoto, Japan), equipped with an ultraviolet (UV)–visible detector (Shimadzu Model SPD10 AV) set at λ 254 nm, was used. The stainless-steel column was a reversed-phase Supelco Ascentis C18 (250 × 4.6 mm, 5.0 µm i.d.) with a Supelguard Ascentis C18 guard column (both from Supelco, Bellefonte, PA, USA). All mobile phases were vacuum filtered through 0.45 μm nylon membranes (Millipore, Burlington, MA, USA). Overall analyses were conducted at 0.5 mL min^−1^. Data acquisition and integration were accomplished by Cromatoplus 2011 software (Messina, Italy). Methanol injections were made every five runs to assess that no carryover occurred.

### 3.5. IAM Chromatography

Phospholipophilicity was experimentally determined using two IAM analytical columns, i.e., IAM.PC.MG (150 × 4.6 mm, 10 μm p.s., pore size: 300 Å) and IAM.PC.DD2 (150 × 4.6 mm, 10 μm p.s., pore size: 300 Å), both from Regis Chemical Company (Morton Grove, IL, USA). The analyses on the IAM.PC.MG were conducted on a LC-20 AD VP (from Shimadzu Corp., Kyoto, Japan), equipped with an ultraviolet (UV)–visible detector (Shimadzu Model SPD10 AV) set at λ = 254 nm. For the determinations on the IAM.PC.DD2, an alliance HPLC system (Waters Corporation, Milford, MA USA) with 2690/2695 separation module, 2996 PDA detector, 717 plus autosampler, and 2487 dual λ absorbance detector, was used at 254 nm wavelength instead. The methods were set, and chromatograms were recorded by Waters Empower^®^ 3 software (Milford, MA USA). On both columns, employed eluents were 0.1 M phosphate buffer at pH 5.0 and acetonitrile at various percentages with a flow rate of 1.0 mL min^−1^. All samples were dissolved in acetonitrile (ca. 10^−4^ M), and chromatographic analyses were carried out at 22 ± 2 °C. The two different IAM stationary phases differ from each other in the end-capping of residual amino groups of the silica-propylamine core, which support C10 and C3 alkyl chains being end-capped by both decanoic and propionic anhydrides (IAM.PC.DD2), while IAM.PC.MG supports hydroxy groups being end-capped by methyl glycolate. IAM parameters performed on both stationary phases have already been found to be strongly interrelated, as assessed in our previous studies [25,26,27,28,31]. The affinity of the chemicals for both IAM.PC.MG and IAM.PC.DD2 was measured as a retention factor extrapolated at 100% of the aqueous phase (kw IAM) by performing a polycratic extrapolation method [52]. Since all the compounds under our investigation required at least the addition of acetonitrile to the mobile phase to elute within 20 min, at least three different mobile phases containing acetonitrile in percentages (φ), ranging from 10% to 30% (*v*/*v*), were employed. All kw IAM values average at least three individual measurements, and the final values are reported as decimal logarithms.

### 3.6. Experimental Permeability Calculation

The skin permeability was calculated as follows: PBs flux (J) was calculated from the quantity of each PB, which permeated through the membrane, divided by the insert membrane surface and the time duration (µg/cm^2^*h). The permeability coefficient (Kp (cm/h)) was determined from J and the drug concentration in the donor phase (Cd (µg/cm^3^)):

J = Qr/A × h(1)

Kp = J/Cd(2)

The ratio of each PB total amount in the receptor fluid compared to the applied amount was calculated to determine the total absorption rate:

Total absorption (%) = Amount receptor fluid/Amount total × 100(3)

### 3.7. Skin Permeation Calculator

For in silico determination of log Kp, the SwissADME^®^ engine (freely accessible at http://www.swissadme.ch (accessed on 27 June 2022) was used, based on a linear model adapted from Potts and Guy, who found that Kp linearly correlated with molecular size and lipophilicity [36].

### 3.8. Statistical Analysis

Calculations were made using Microsoft^®^ Excel^®^ of Office 365. All data sets are shown as the mean ± standard deviation (SD), with statistically significant differences determined by a *t*-test with probability (*p*) values < 0.05.

## 4. Conclusions

We studied the affinity of PBs for biomembranes, which plays a crucial role in their membrane barrier passage that strongly affects their toxicokinetic. The study of the permeation of PBs in a formulation vehicle was not in the scope of this work, as whichever experimental design would have never mirrored the plethora of cosmetic and pharmaceutical formulations in which PBs can be found. The actual issue of PBs’ toxicity is their occurrence, even at low regulated doses, in many consumer goods of large consumption that can create continuous and multiple sources of exposure for humans and generate a chronic toxicity issue.

Our results demonstrate that caution is required when using in silico and chromatographic data, with the aim of surrogating in vitro permeation values. Indeed, IAM data reflect the affinity of compounds for phospholipids, but not necessarily their permeation through complex barrier. In silico data are based on the application of mathematical models that are often based in full or in part on molecular lipophilicity. However, their performance can unfortunately be substandard when passage is modulated by other features.

## Figures and Tables

**Table 1:** Chromatographic and physicochemical parameters of the eight chemicals.

Compound	S (mg L^−1^)	Molecular Weight (g/mol)	Log P	Range (µg mL^−1^)	Mobile Phase Composition ACN:H_2_O	Rt (min)	Slope	Intercept	r^2^
pHBA	5.00 × 10^3^	138.12	1.58	20–40	40:60 *	6.1	43879	11,792	0.9992
MP	2.50 × 10^3^	152.16	1.96	5–40	50:50	8.3	402.09	1120.1	0.9988
EP	8.85 × 10^2^	166.18	2.47	5–40	50:50	10.9	3503.5	1344.3	0.9861
PrP	5.00 × 10^2^	180.21	1.96	2.5–20	50:50	15.7	1059.8	1647.2	0.9973
iPrP	5.00 × 10^2^	180.21	1.96	2.5–20	60:40	10.6	795.67	2048.8	0.9975
BuP	2.07 × 10^2^	194.23	3.57	2.5–20	60:40	14.5	2275.3	2863.3	0.9256
iBuP	2.07 × 10^2^	194.23	3.57	2.5–20	50:50	23.0	1271.4	1831.0	0.9984
BzP	0.92 × 10^2^	228.25	3.56	2.5–20	60:40	14.5	723.07	2675.1	0.9976

S = Solubility in water; Log P were taken from PubCHEM; * the aqueous phase was phosphate buffer pH 3.0.

**Table 2:** Permeability coefficient and maximum flux ± standard deviation of the three compounds able to cross the skin. Median of % permeation, with 95% interval confidence given in parentheses.

Compound	Maximum Flux (µg/cm^2^/h)	Kp (cm/h)	Permeation (%) (Median)
pHBA	12.68 ± 4.08	0.012	0.997 (0–1.22)
MP	76.23 ± 26.60	0.305	9.148 (4.94–19.70)
EP	2.35 ± 0.71	9.4 × 10^–4^	0.110 (0–0.11)
PrP	Nd	nd	nd
iPrP	Nd	nd	nd
BuP	Nd	nd	nd
iBuP	Nd	nd	nd
BzP	Nd	nd	nd

**Table 3:** Experimental phospholipophilicity values measured on both the IAM.PC.MG and IAM.PC.DD2 stationary phases.

Compound	logk_w_ ^IAM.MG^	logk_w_ ^IAM.DD2^
pHB	−0.955	−1.054
MP	0.615	1.306
EP	0.956	1.751
PrP	1.339	2.267
iPrP	1.227	1.914
BuP	1.818	2.812
iBuP	1.708	2.448
BzP	2.292	2.735

**Table 4:** Logarithm of experimental and calculated permeability coefficient (Kp). Log Kp * = Determined in the present study; Log Kp ** = SwissADME calculator.

Compound	Log Kp * cm/s	Log Kp ** cm/s
pHBA	−5.48	−6.02
MP	−4.07	−5.84
EP	−6.58	−5.56
PrP	Nd	−5.24
iPrP	Nd	−5.40
BuP	Nd	−4.95
iBuP	Nd	−5.05
BzP	Nd	−4.93

## Data Availability

Not applicable.

## Supplementary Materials

The following supporting information can be downloaded at: https://www.mdpi.com/article/10.3390/molecules27134263/s1, Figure S1: Relationship between calculated phospholipophilicity data (clog kw IAM.MG and clog kw IAM.DD2) for both stationary phases achieved by Web service (available at https://nova.disfarm.unimi.it/vegaol/logkwiam.htm.); Table S1: Chromatographic Limit of Detection (LOD) of the seven analyzed parabens and their metabolite; Table S2: Phospholipophilicity calculated for both stationary phases (clog kw IAM.MG**, clog kw IAM.DD2**) in silico, via a user-friendly tool Web service (available at https://nova.disfarm.unimi.it/vegaol/logkwiam.htm.), able to predict phospholipophilicity of any molecule included in the PubChem collection.
