# Supplementary material for: Parabens Permeation through Biological Membranes: A Comparative Study Using Franz Cell Diffusion System and Biomimetic Liquid Chromatography
Source: Molecules. 2022 Jul 1;27(13):4263. doi: 10.3390/molecules27134263 (PMC9268571; doi:10.3390/molecules27134263)
Supplement: Supplementary file 1 [file molecules-27-04263-s001.zip › molecules-1784402-supplementary.pdf]

# Supplementary Materials

# Parabens Permeation through Biological Membranes: A Comparative Study Using Franz Cell Diffusion System and Biomimetic Liquid Chromatography

**Table S1.** Chromatographic Limit of Detection (LOD) of the seven analyzed parabens and their metabolite.

**Table S2.** Phospholipophilicity calculated for both stationary phases (clog $k_w$ $^{IAM.MG**}$, clog $k_w$ $^{IAM.DD2**}$) in silico.

**Figure S1.** Relationship between calculated phospholipophilicity data.

**Table S1.** Chromatographic Limit of Detection (LOD) of the seven analyzed parabens and their metabolite.

| Compound | LOD µg $mL^{-1}$ |
|---|---|
| pHBA | 0.886 |
| MP | **0.810** |
| EP | 0.052 |
| iPrP | 0.129 |
| PrP | 0.213 |
| iBuP | 0.109 |
| BuP | 0.312 |
| BzP | 0.187 |

**Table S2.** Phospholipophilicity calculated for both stationary phases (clog $k_w$ $^{IAM.MG}$** , clog $k_w$$^{IAM.DD2}$**) in silico, via a user-friend tool Web service (available at https://nova.disfarm.unimi.it/vegaol/logkwiam.htm.), able to predict phospholipophilicity of any molecule included in the PubChem collection.

| Compound | clog $k_w$ $^{IAM.MG}$ | clog $k_w$ $^{IAM.DD2}$ |
|---|---|---|
| pHBA | 0.277 | 0.354 |
| MP | 0.499 | 0.737 |
| EP | 0.716 | 1.000 |
| PrP | 0.988 | 1.334 |
| iPrP | 0.957 | 1.314 |
| BuP | 1.282 | 1.698 |
| iBuP | 1.216 | 1.630 |
| BzP | 1.611 | 1.941 |

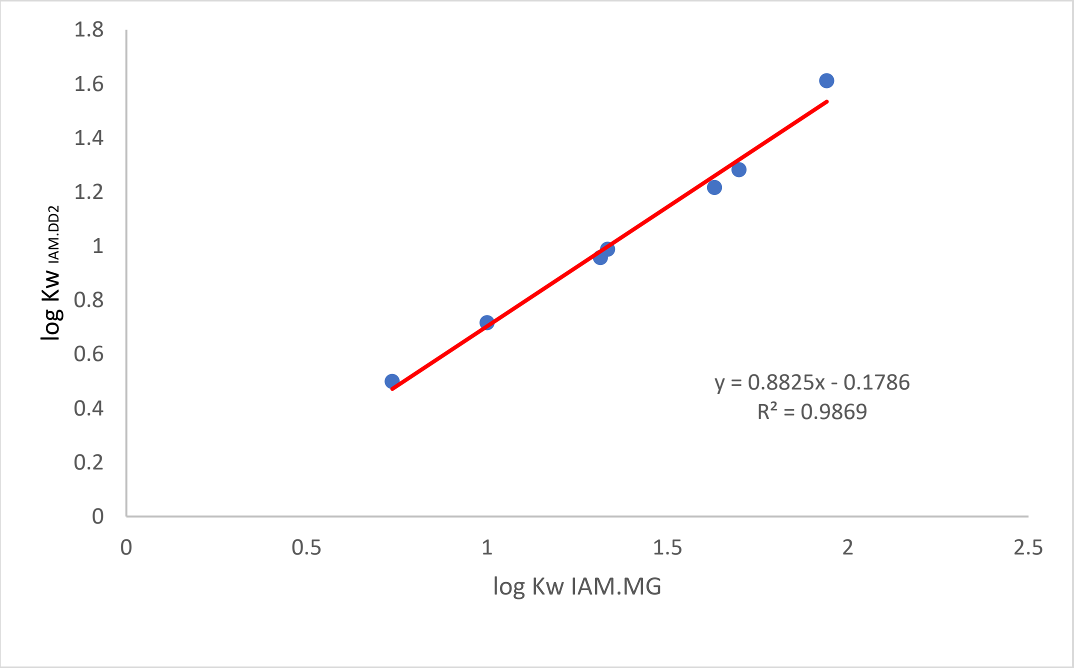


**Figure S1.** Relationship between calculated phospholipophilicity data (clog $kw^{IAM.MG}$ and clog $kw^{IAM.DD2}$) for both stationary phases achieved by Web service (available at https://nova.disfarm.unimi.it/vegaol/logkwiam.htm.).
